# Variability of Bio-Clinical Parameters in Chinese-Origin Rhesus Macaques Infected with Simian Immunodeficiency Virus: A Nonhuman Primate AIDS Model

**DOI:** 10.1371/journal.pone.0023177

**Published:** 2011-08-05

**Authors:** Song Chen, Chunhui Lai, Xiaoxiang Wu, Yaozheng Lu, Daishu Han, Weizhong Guo, Linchun Fu, Jean-Marie Andrieu, Wei Lu

**Affiliations:** 1 Sino-French Collaboration Center for AIDS Research and Department of Cell Biology, Peking Union Medical College and Chinese Academy of Medical Science, Tsinghua Universtiy, Beijing, China; 2 IRD-UMI233 China Branch/Tropical Medicine Institute, Guangzhou University of Chinese Medicine, Guangzhou, China; 3 Institut de Recherche sur les Vaccins et l'Immunothérapie des Cancers et du Sida at the Saints-Pères Biomedical Center, Université Paris Descartes, Paris, France; University of Rochester, United States of America

## Abstract

**Background:**

Although Chinese-origin Rhesus macaques (Ch RhMs) infected with simian immunodeficiency virus (SIV) have been used for many years to evaluate the efficacy of AIDS vaccines and therapeutics, the bio-clinical variability of such a nonhuman primate AIDS model was so far not established.

**Methodology/Principal Findings:**

By randomizing 150 (78 male and 72 female) Ch RhMs with diverse MHC class I alleles into 3 groups (50 animals per group) challenged with intrarectal (ir) SIVmac239, intravenous (iv) SIVmac239, or iv SIVmac251, we evaluated variability in bio-clinical endpoints for 118 weeks. All SIV-challenged Ch RhMs became seropositive for SIV during 1–2 weeks. Plasma viral load (VL) peaked at weeks 1–2 and then declined to set-point levels as from week 5. The set-point VL was 30 fold higher in SIVmac239 (ir or iv)-infected than in SIVmac251 (iv)-infected animals. This difference in plasma VL increased overtime (>100 fold as from week 68). The rates of progression to AIDS or death were more rapid in SIVmac239 (ir or iv)-infected than in SIVmac251 (iv)-infected animals. No significant difference in bio-clinical endpoints was observed in animals challenged with ir or iv SIVmac239. The variability (standard deviation) in peak/set-point VL was nearly one-half lower in animals infected with SIVmac239 (ir or iv) than in those infected with SIVmac251 (iv), allowing that the same treatment-related difference can be detected with one-half fewer animals using SIVmac239 than using SIVmac251.

**Conclusion/Significance:**

These results provide solid estimates of variability in bio-clinical endpoints needed when designing studies using the Ch RhM SIV model and contribute to the improving quality and standardization of preclinical studies.

## Introduction

The nonhuman primate (NHP) models have been used for more than two decades to evaluate HIV-1 vaccine candidates worldwide. So far, no effective vaccine is available for preventing or controlling HIV-1 infection. Due to the lack of clarity about what host immune responses are required to prevent HIV-1/SIV infection or to control viral replication/protect against disease progression, the efficacy of prevention of viral infection or protection of disease progression following experimental SIV challenge of NHPs vaccinated with a prototype SIV vaccine is now being reconsidered as the primary criterion to conclude go/no-go decision prior to entry into phase I clinical trial [1], [2]. Since the HIV-1 does not replicate in most animal species hitherto tested, including rodents and small non-human primates, SIV-HIV chimera (SHIV) has been constructed by inserting partial genome of HIV-1 into SIV and applied to infect rhesus monkeys as a mimic animal model of HIV/AIDS ten years ago [3]. However, the reliability of SHIV model has recently been doubted, since an SIV version of the Merck Ad5 HIV-1 gag vaccine showed to be effective in SHIV model [4] but proved to be ineffective for protecting human from infection in the STEP clinical trials [5]. Interestingly, it has been shown after the human trials of the HIV-1 vaccine that the SIV version of the Merck Ad5 HIV-1 gag vaccine was also ineffective in reducing post-infection viral load of vaccinated rhesus macaques after SIVmac239 challenge [6]. On the other hand, some prototype SIV vaccines have been showed to be only effective at reducing post-infection viral load in macaques with a specific MHC class I allele, Mamu A*01 [7]. Furthermore, certain MHC-1 alleles (such as Mamu A*01, Mamu B*08, and Mamu B*17) might be associated with an increased capacity to control post-infection viral load in the absence of immunization [8], [9], [10], [11]. Therefore, the uses of appropriate SIV strains and animals with diverse MCH-I alleles are critical for predicting the outcome of a human clinical trial in cost-effective and resource-efficient ways.

Up-to-date, SIV-infected rhesus macaques of Indian (In RhMs) origin are among the most widely used NHP models for HIV/AIDS. Although SIV-infected rhesus macaques of Chinese origin (Ch RhMs) have also been used in the field for many years [12], [13], [14], [15], due to the relatively small size of animal numbers in each individual study, it is very hard to define a standardized model system with statistically meaningful parameters for the evaluation or preclinical testing of candidate vaccines. In present study, we undertook a prospective study to evaluate the virological, immunological, and clinical evolutions of Ch Rh population infected with SIVmac239 or SIVmac251 (the 2 frequently used SIV strains) through different (intravenous versus intrarectal) routes of viral challenge, aimed at developing a standardized model system for any independent or coordinated NHN studies in the field.

## Results

### MHC class I alleles in Ch RhMs

**Figure 1:**
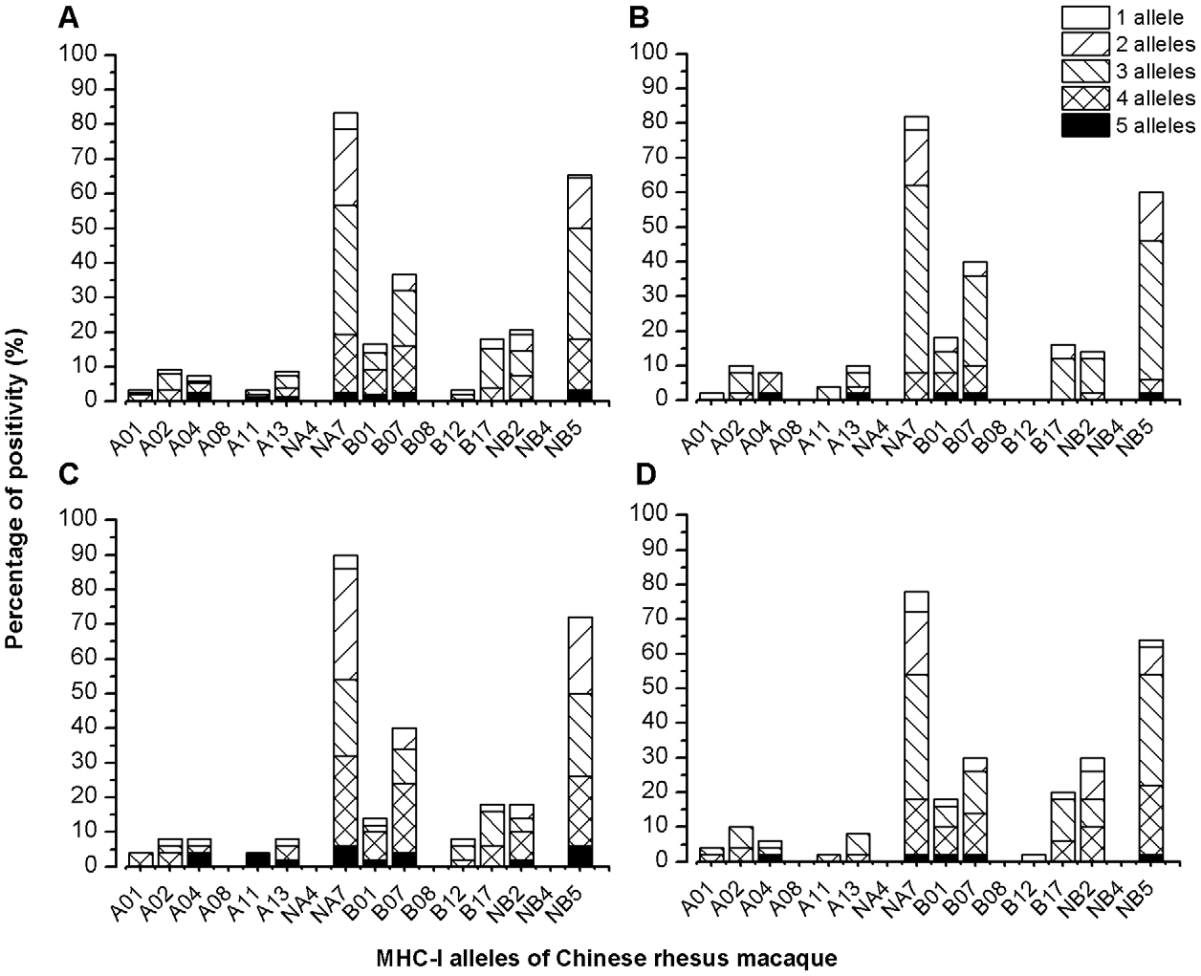
Distribution of MHC class I alleles (including patterns of shared alleles) by the sequence-specific primers (SSP)-PCR assay in the whole 150 Ch RhMs (A), 50 ir SIVmac239-infected Ch RhMs (B), 50 iv SIVmac239-infected Ch RhMs (C), or 50 iv SIVmac251-infected Ch RhMs (D). Note that the 3 out of 150 (2%) samples were negative for the SSP-PCR assay.

We started to genotype the MHC class I alleles using the SSP-PCR assay in the PBMC samples taken from 150 Ch RhMs. Twelve (A01, A02, A04, A11, A13, NA7, B01, B07, B12, B17, NB2, and NB5) out of the 16 included RhM MHC-I alleles were detected in Ch RhMs. The remaining 4 MHC-I alleles (A08, NA4, B08, and NB4) were not detected in the 150 Ch RhMs. Of the 150 Ch RhM samples, 147 (98%) were found to have a single MHC-I allele or multiple (2–5) shared *Mamu-A* and/or *Mamu-B* alleles (Fig. 1a). Due to the complexity of Ch RhM MHC-I alleles, we decided to distribute the animals to each group of the experiments by randomization. The animals were then challenged with intrarectal (ir) 10^5^ TICD_50_ SIVmac239 (n = 50) (Fig. 1b) or with intravenous (iv) 200 TICD_50_ SIVmac239 (n = 50) (Fig. 1c) or 200 TICD_50_ SIVmac251 (n = 50) (Fig. 1d).

### Antibody responses in SIV-infected Ch RhMs

**Figure 2:**
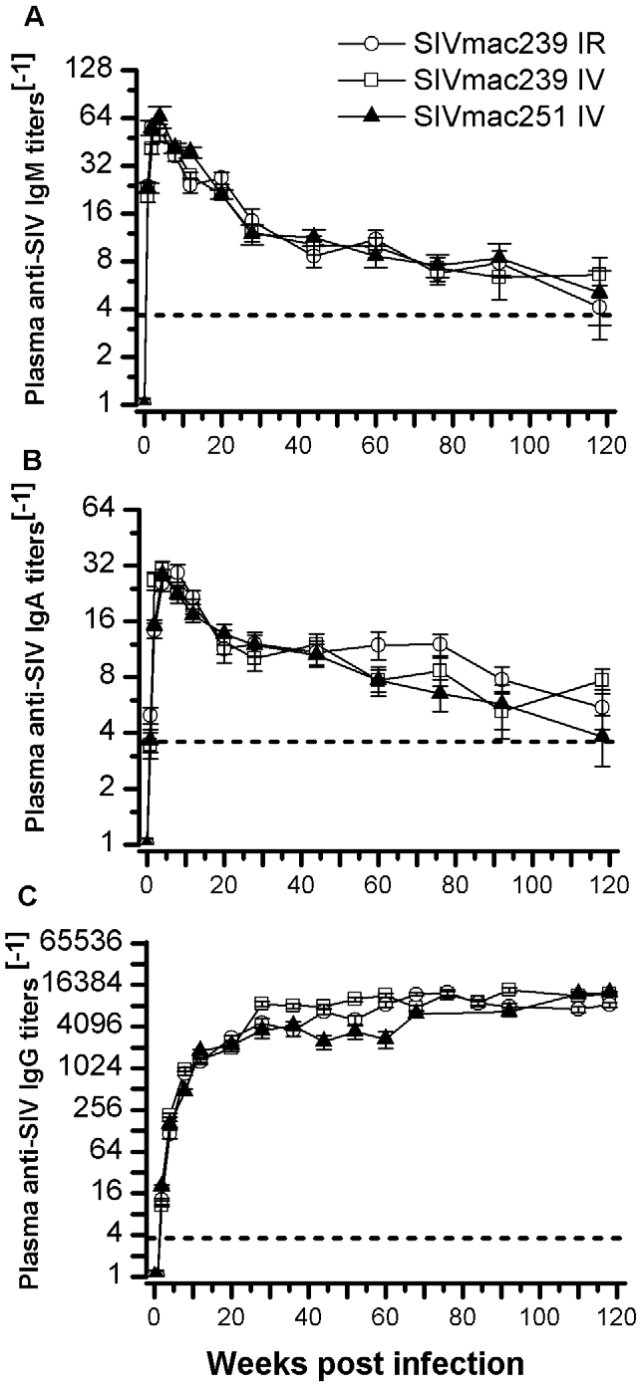
Humoral immune responses in Ch RhMs randomly challenged with pathogenic SIVmac239 (ir or iv) or SIVmac251 (iv). (A) Anti-SIV IgM antibody titers (mean ± SD) in plasma following 118 weeks post viral challenge. (B) Anti-SIV IgA antibody titers (mean ± SD) in plasma following 118 weeks post viral challenge. (C) Anti-SIV IgG antibody titers (mean ± SD) in plasma following 118 weeks post viral challenge.

As expected, all 150 Ch RhMs became seropositive for SIV 1–2 weeks after SIV challenges. The peak titers of plasma anti-SIV antibodies were weeks 2–3, weeks 4–8, and after 28 weeks for IgM, IgA, and IgG respectively. No significant difference in plasma anti-SIV antibody titers was observed between animals randomly challenged with SIVmac239 (ir or iv) or SIVmac251 (iv) (P>0.1 by Mann-Whitney) (Fig. 2a–c).

### Disease progression in SIV-infected Ch RhMs

**Figure 3:**
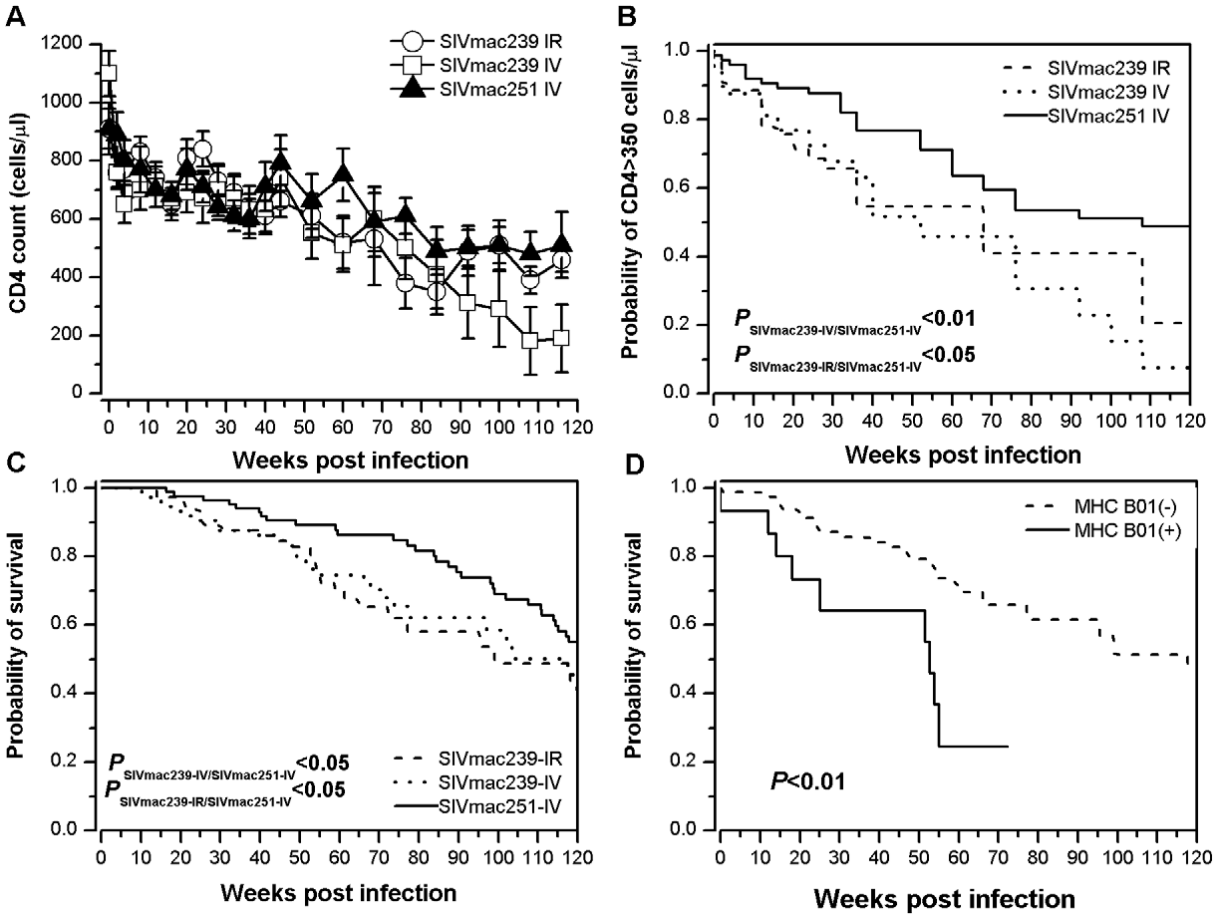
CD4+ T-cell counts and animal survivals in Ch RhMs randomly challenged with pathogenic SIVmac239 (ir or iv) or SIVmac251 (iv). (A) Evolution of CD4^+^ T-cell counts (mean ± SD) following 118 weeks post viral challenge. (B) Kaplan-Meier plot of probability for maintaining a stable CD4^+^ T-cell count following 118 weeks post viral challenge. (C) Kaplan-Meier plot of survival probability of Ch RhMs following 118 weeks post viral challenge. (D) Survival curve of SIVmac239-infected Ch RhMs with or without MHC-I B01 allele.

CD4^+^ T-cell counts declined rapidly during the first 4 weeks post-infection and decreased progressively thereafter in the 3 groups of animals (Fig. 3a). Kaplan-Meier analysis of the probability of SIV-infected animals maintaining a CD4^+^ T-cell count over 350 cells/µl demonstrated that significant lower probabilities to maintain a stable CD4^+^ T-cell count were observed in animals challenged with either intrarectal SIVmac239 (20%; T_50_: 52 weeks) (P<0.05) or intravenous SIV mac239 (8%; T_50_: 68 weeks) (P<0.01) as compared to animals challenged with intravenous SIVmac251 (48%; T_50_: 108 weeks) (Fig. 3b).

Kaplan-Meier survival analysis showed that animals challenged with ir or iv SIVmac239 had lower survival rates (34% or 36% at week 118) with a median survival time (T_50_) of 99 weeks for ir SIVmac239 (95% confidence interval between 69 and 129) or 117 weeks for iv SIVmac239 (95% confidence interval between 101 and 134) as compared to those of animals challenged with intravenous SIVmac251 (54% at week 118) with a T_50_ of >120 weeks (P<0.05) (Fig. 3c). Sixteen MHC-I B01-positive animals infected with SIVmac239 were found to be associated with a poorer survival (T_50_ of 54 weeks and 95% confidence interval between 51 and 57) than those animals that were negative for MHC-I B01 allele (T_50_ of 118 weeks and 95% confidence interval between 92 and 144) (P<0.01) (Fig. 3d). A total of 88 animals (33, 32, and 23 for intrarectal SIVmac239, intravenous SIVmac239, and intravenous SIV251 respectively) died from AIDS-defining illness between 26 and 118 weeks post infection.

### Plasma viral loads in SIV-infected Ch RhMs

**Figure 4:**
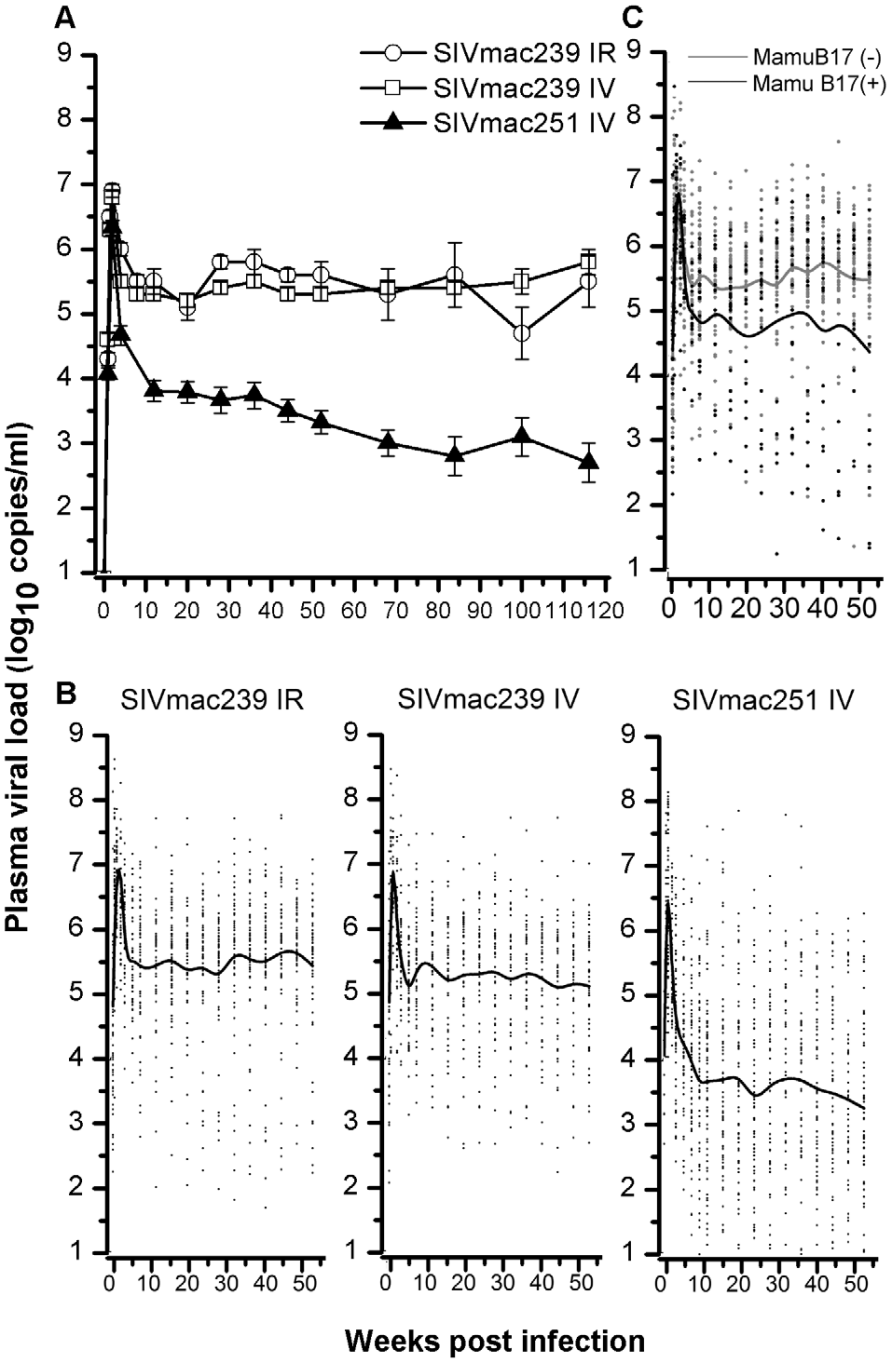
Plasma SIV RNA viral loads in Ch RhMs randomly challenged with pathogenic SIVmac239 (ir or iv) or SIVmac251 (iv). (A) Evolution of plasma SIV RNA viral loads (geometric mean ± SE) following 118 weeks post viral challenge. (B) Smoothed curve of the SIV RNA levels in plasma of animals following 52 weeks post viral challenge with SIVmac239 (ir or iv) or SIVmac251 (iv). (C) Smoothed curve of the SIV RNA levels in plasma of SIVmac239-infected Ch RhMs with or without MHC-I B17 allele.

The peaks of plasma viral loads (1–2 weeks) were similar between these 3 groups of animals. The set-point viral loads were around 30 fold lower in animals challenged with SIVmac239 (ir or iv) as compared to those of animals challenged with SIVmac251 (iv) (P<0.01) and this difference increased to more than 100 fold as from week 68 (P<0.01) (Fig. 4a). A more pronounced variation in the set-point viral loads was observed in animals challenged with intravenous SIVmac251 as compared to those of animals challenged with intrarectal/intravenous SIVmac239 (P<0.01) (Fig. 4b). Seventeen MHC-I B17-positive animals infected with SIVmac239 were found to be associated with a lower set-point viral load (nearly 10 fold) than those negative for MHC-I B17 allele (P<0.01) (Fig. 4c). We observed 4% and 6% of animals with a persistent low viral load (<1000 copies/ml) in the intravenous and intrarectal SIVmac239 challenge groups respectively, while this figure was 28% in the intravenous SIVmac251 challenge group.

### Sample size estimations using SIV-infected Ch RhM models

Having observed the distinct variations in the set-point viral loads between animals challenged with SIVmac239 (ir or iv) or SIVmac251 (iv), we performed statistical power calculation and sample size estimation for Ch RhM AIDS model systems using the peak viral load and the set-point viral load as endpoints. The peak viral load was defined as the maximum viral load measured between weeks 1–2 inclusive after SIV challenge for each animal while the set-point viral load was defined as the average value for an animal from weeks 5–10, as described previously [16]. To estimate the variability for each of the 2 commonly used plasma viral load measures, the overall standard deviation (SD) was calculated for each of the 3 experimental groups. SD for peak viral loads were similar for the 2 modes of SIVmac239 challenge (0.48 log_10_ SIV RNA copies/ml for ir and 0.50 log_10_ SIV RNA copies/ml for iv), same as for set-point viral loads (0.85 log_10_ SIV RNA copies/ml for ir and 0.79 log_10_ SIV RNA copies/ml for iv). In contrast, SD for peak or set-point viral loads were substantially higher in animals challenged with intravenous SIVmac251 (0.76 log_10_ SIV RNA copies/ml and 1.14 log_10_ SIV RNA copies/ml respectively) as compared to SIVmac239 (ir or iv). These variability estimates were then used to calculate the sample size design using a commercial Power Analysis and Sample Size software (PASS version 2008), assuming a two-group experiment with groups of equal size and variability across groups. Due to a lower variability, experiments using SIVmac239 (ir or iv) require few animals than experiments using SIVmac251 to detect the same difference. For example, 4/8 animals per group would be required to detect a difference of 1.5 log_10_ SIV RNA copies/ml for peak/set-point viral load between the two groups with 90% power for either intrarectal or intravenous SIVmac239. Nearly twice as many animals per group (7/14) would be required to detect the same difference for peak/set-point viral loads using SIVmac251 (Table 1).

**Table 1:** Sample size requirements for each group with different powers and viral load (VL) measures.

Infecting virus	Measures	DD^a^ and power	SD	Sample size using estimated SD				
SIVmac239-IV	Peak VL(1–2 weeks)	**DD**		**1**	**1.25**	**1.5**	**1.75**	**2**
		80%	0.48	5	4	4	3	3
		90%		6	5	4	3	3
	Set-point VL(5–10 weeks)	**DD**		**1**	**1.25**	**1.5**	**1.75**	**2**
		80%	0.85	13	9	7	5	5
		90%		17	11	8	7	5
SIVmac239-IR	Peak VL(1–2 weeks)	**DD**		1	1.25	1.5	1.75	2
		80%	0.50	6	4	4	3	3
		90%		7	5	4	4	3
	Set-point VL(5–10 weeks)	**DD**		**1**	**1.25**	**1.5**	**1.75**	**2**
		80%	0.79	11	8	6	5	4
		90%		15	10	7	6	5
SIVmac251-IV	Peak VL(1–2 weeks)	**DD**		**1**	**1.25**	**1.5**	**1.75**	**2**
		80%	0.76	11	7	6	5	4
		90%		14	9	7	6	5
	Set-point VL(5–10 weeks)	**DD**		**1**	**1.25**	**1.5**	**1.75**	**2**
		80%	1.14	22	15	11	8	7
		90%		29	19	14	10	8

a Mean detectable difference (DD) in log_10_ SIV RNA copies/ml between experimental and control groups.

## Discussion

Rhesus macaques of Indian and Chinese origin are commonly used today in the animal models for studying a variety of human diseases and vaccines. Ch RhMs differ from their Indian counterparts in morphology and physiology [17], [18], as well as in their genetics [19], [20], [21], [22]. Historically, In RhMs have been used more frequently than Ch RhMs in research. However, there is a trend of increasing use of Ch RhMs worldwide due to the long-lasting embargo (since 70 s) on export of RhMs from India. Previous SIV NHP studies have shown marked differences in disease progression between Chinese- and Indian-origin macaques [12], [13], [14], [15]. In the present study (with a large number of animals), we showed that randomized protocols for the use of Ch RhM SIV model system could be standardized for testing AIDS vaccines or therapeutics.

Although a previous study has reported no difference in survival in In RhMs infected with either SIVmac239 or SIVmac251 [23], we did observe that Ch RhMs infected with SIVmac239 by either the intrarectal or intravenous route had higher viral loads than SIVmac251 and progressed to AIDS more rapidly. This discrepancy could results from the distinct MHC genes between In and Ch RhMs [19], [20], [21], [22]. In contrast to the molecularly cloned SIVmac239 [24], the swarm (uncloned) SIVmac251 might raise a more broad anti-viral immunity against the SIVmac251 quasispecies in Ch RhMs to control viral load and to slow disease progression. The fact that SIVmac239 (ir or iv)-infected Ch RhMs had a higher and less variable set-point viral load and a more rapid rate of progression to AIDS or death as compared to SIVmac251-infected Ch RhMs (Figs. 3 and 4), implies a more advantage of using SIVmac239-infected Ch RhM model in terms of shorter time for achieving the clinical endpoints and smaller sample size requirement for detect the same difference. Importantly, such a SIVmac239-infected Ch RhM model system is also comparable to the most frequently used SIVmac251-infected In RhMs in terms of variability in the peak and set-point plasma viral loads measurements [16]. The similar estimates of variability for peak and set-point plasma viral loads as well as for clinical endpoints we observed between intrarectal and intravenous SIVmac239 suggest that SIV replicates in an overall similar lymphoid compartment regardless the entry routes of infecting virus. Although it has been suggested that humans infected with HIV-1 through different routes of viral transmission (such as heterosexual or homosexual contact, needle-sharing among injecting drug users, or blood transfusion) might have different rates of disease progression [25], [26], the reasons for such differences have not-yet been clarified.

Previous studies using SIVmac239 in Ch RMs have reported the elite controllers (EC: animals who had low viral loads for a time-period of >5 years) in nearly 1/3 of animals [27], while we observed only 4–6% of animals with a persistent low viral load (<1000 copies/ml) in the intravenous and intrarectal SIVmac239 challenge groups. It is worth to note that the EC prevalence in HIV-1-infected human population is <0.5% [28]. Although the cause for such a major discrepancy in the EC prevalence between the present study and previous ones remains unknown, it might be however plausible that such a discrepancy could result from distinct subspecies of Ch RMs employed in different studies (a subspecies of Guangxi was used in our study) since the complex subspecies of Ch RMs have been indeed documented in China [29], [30].

Rhesus macaque MHC-1 alleles Mamu A*01, Mamu B*08, and Mamu B*17 have been suggested to be associated with an increased capacity to control post-infection viral load in In RhMs [8], [9], [10], [11]. We observed that the MHC-1 Mamu B*08 allele was absence in the 150 Ch RhMs and low frequencies of Ch RhMs were positive for Mamu A*01 (3.3%) and Mamu B*17 (18%). Indeed, the Mamu B*17 allele was found to be associated with a significant lower set-point viral load in SIVmac239-infected Ch RhMs as compared to those animals negative for Mamu B*17 (Fig. 4c), although no difference in survival was observed in animals positive or negative for Mamu B*17 (data not shown). In addition, we observed also that Mamu B*01 was associated with a poorer survival (Fig. 3d), but no difference in viral load was observed in SIVmac239-infected Ch RhMs positive or negative for Mamu B*01 (data not shown). An early study has reported that Mamu B*01 was associated with higher viral load in In RhMs infected by SIVmac251 [31]. These discrepancies between viral load and survival endpoints regarding to defined macaque MHC-1 alleles are most likely caused by a relatively low sensitivity of the sequence-specific primers (SSP) PCR assays since one or two site-mutation might switch off the outcome in a PCR-based assay. Thus, further analysis by sequencing the full macaque MHC-1 genes should be required for establishing the potential link between MHC-1 genotpyes/amino acid sequences and viro-immunological makers in SIV-infected macaques. Nevertheless, duo to the complexity of Ch RhM MHC-I molecules with multiple shared alleles (Fig. 1), randomization of animals would be among the most cost-effective and resource-efficient ways for evaluating the efficacy of an experimental vaccine or drug in the animal model system.

Given that large clinical trials aimed at inducing conventional HIV-1-specific antibodies and/or CTL responses have failed to show a significant clinical efficacy [6], [32], it is now necessary to search new alternative approaches for combating against AIDS [33]. More recently, monoclonal antibodies directed against the CD4-binding site of gp120 have been reported to be promising to neutralize 90% of circulating HIV-1 isolates. However, whether such broad neutralizing antibodies might be generated in vivo for protecting people from either mucosal or systematic infection should be further evaluated in the Ch or In RhM model system before the start of a large human trial.

## Materials and Methods

### Animals

Colony-bred rhesus macaques (*Macaca mulatta*) of Chinese origin (Ch RhM) (a subspecies of Guangxi) were housed at the non-human primate laboratory of Gaoyao Experimental Animal Center (Guangdong, China) in accordance with the regulations of the National Institutes of Health ‘Guide for the Care and Use of Laboratory Animals’ and all details of animal welfare and steps taken to ameliorate suffering were in accordance with the recommendations of the Weatherall report, “The use of non-human primates in research”. Briefly, animals were housed in a clean room (class D) with well-controlled temperature (20–25°C) and humidity (30–70%). All parameters (temperature, humidity, and pressure) were monitored in real time by a computer-based continuous recording system. Each clean room had adequately lighting and was equipped with 24 surrounding cages allowing the animals have sight and sound of each other. The cage size was 1.2 m (length)×1.2 m (wide)×1.2 m (height) which had almost 2 fold more space than the minimum space recommended for a group of nonhuman primate with a weight up to 10 kg by the Institute for Laboratory Animal Research, National Research Council of National Academy (USA) [34]. For the purpose of best practice in environmental enrichment, each room was installed with a loudspeaker providing 4 hours' music per day (2 hours in the morning and 2 hours in the afternoon) and each cage was supplied with toys. Moreover, we implanted regular staff training programs in order to ensure good standards of welfare. For instance, improving the efficacy of anesthetic practices to prevent animals from any unnecessary injury or training animals to present a leg voluntarily for collecting a blood sample so as to avoid anesthesia.

All animals were in good health, 3–6 years old, weighed 4–8 kg and were seronegative for SIV, SRV, simian T cells lymphotropic virus 1, hepatitis B virus, and Herpesvirus simiae (B virus). X ray and skin test (PPD) were performed at entry for all animals to exclude potential carriers of tuberculosis. This study was carried out in strict accordance with the recommendations in the Guide for the Care and Use of Laboratory Animals of the National Institute of Health. The protocol was approved by the Committee on the Ethics of Animal Experiments of the Guangzhou University of Chinese Medicine (Permit number: 15-0507). A total of 150 (78 males and 72 females) Ch RhMs were recruited for this prospective study. To better control the bias, we assigned first the 78 males and then the 72 females to 3 groups using the computerized Random Number Generator Program (randnum.exe) by adding the constraint of equal sample sizes after the random numbers are distributed (i.e. 26 and 24 for males and females respectively).

### MHC class I typing

The rhesus macaque MHC class I alleles of 150 recruited Ch RhMs were genotyped in peripheral blood monoculear cells (PBMC) samples using sequence-specific primers (SSP) PCR assays for representative *Mamu-A* and *Mamu-B* sequences as previously described [8], [10].

### SIV challenges

SIVmac251 (3.2×10^3^ TCID_50_/ml) and SIVmac239 (5×10^5^ TCID_50_/ml) viruses (gifts of P.A. Marx in 1994) were propagated in vitro using primary macaque PBMC and the first passages of the original viral stocks were used for challenge. Briefly, PBMC were isolated from whole blood of healthy Ch RhMs. After depleting CD8 positive cells by MACS CD8 MicroBead (Miltenyi Biotec, Auburn, CA) according to the manufacturer's recommendations, the cells were stimulated with 0.5 µg/mL staphylococcal Enterotoxin B (SEB) (Sigma, St Louis, Missouri) for 3 days in RPMI 1640 containing 10% fetal bovine serum and 50 IU/mL rhIL-2. The CD8-depleted/SEB-stimulated PBMC pooled from 20 Ch RhMs were inoculated with SIVmac251 or SIVmac239 at 10^−3^ MOI for 2 hours, and were then cultured at a density of 10^6^/mL in RPMI 1640 containing 10% fetal bovine serum and 50 IU/mL rhIL-2. Half culture medium was replaced with the fresh culture medium every other day, and cell-free supernatants between 6 and 20 days post inoculation were collected. After the measurements of 50% tissue-cell infectious doses (TCID_50_) titrated on CEM174 cells and viral loads assayed by real-time PCR, the pooled supernatants collected between 6 and 20 days were aliquoted and stored at −80°C until to use. The infectious doses of viral stocks used in this study were 2×10^5^ TCID_50_/mL (2.3×10^8^ copies/mL) for SIVmac251 and 5×10^5^ TCID_50_/mL (3.4×10^8^ copies/mL) for SIVmac239.

The animals (n = 150) were randomly assigned into the 3 experimental groups: 1) challenged with intrarectal SIVmac239 (10^5^ TICD_50_, n = 50); 2) challenged with intravenous SIVmac239 (200 TICD_50_, n = 50); and 3) challenged with intravenous SIVmac251 (200 TICD_50_, n = 50). Since SIVmac239 was cloned from SIVmac251, we thought initially to compare the intravenous versus intrarectal challenge of SIVmac239 for promoting the use of molecularly cloned viral strain in order to reduce the bio-variability in the NHP AIDS models. The intravenous uncloned SIVmac251 challenge alone was included as a parallel control. Before intrarectal inoculation, macaques were submitted to 40 hours of hydric diet (overnight plus whole day). Then, animals were placed in a sternal position with the pelvis propped up at an approximately 45° angle after being anesthetized (10 mg/kg of body weight ketamine i.m. and 0.5 mg/kg xylazine i.m.). A lubricated 2.5-mm-outer-diameter pediatric nasogastric-feeding tube was inserted gently into the rectum of the animal approximately 8 cm with caution so as to prevent mucosa trauma. One ml of 1×10^5^ TCID_50_/mL virus was injected through the catheter, followed by 2-ml air to push residual virus out without viral dilution. The animal was kept tilted at a 45° angle and remained immobile for at least 20 min after inoculation. For intravenous challenge, 1 mL properly diluted virus were injected into saphenous vein of anesthetized animals. The animals were then followed up for 118 weeks thereafter. The dead animals were diagnosed by autopsy as AIDS-defining illnesses (such as opportunistic infections, lymphoma, meningitis or encephalitis).

### Assay for antibodies

Anti-SIV IgG, IgM, and IgA antibodies in plasma were titrated by an immunofluorescence antibody (IFA) assay [35], using FITC-conjugated goal anti-macaque IgG (Sigma), IgM (ADI, San Antonio, Texas), or IgA (ADI) antibodies. The sensitivity of IFA assay was a titer of 8 for IgG and a titer of 4 for IgM and IgA.

### Flow cytometry

Flow-cytometry analysis was carried out with FACScalibur (BD Biosciences, San Jose, California) using fluorescence-labeled monoclonal antibodies: CD3-PE-Cy7 (clone SP34-2), CD4-PE (clone MT477), and CD8-PerCP (clone RPA-T8) (BD Biosciences).

### Viral loads

Plasma SIV RNA was quantified by a quantitative RT-PCR with primers (sense, 5′-GAGGAAAAGAAATTT GGAGCAGAA-3′; antisense, 5′-GCTTGATGGTCTCCCACACAA-3′) and probe (5′-FAM-AAAGTTGCACCCCCTATGACATTAATCAGATGTTA-TAMRA-3′) using a 7500 Fast Real-Time PCR System (Applied Biosystems, Foster City, California, USA). The sensitivity of quantitative RT-PCR was 10 copies for the input SIV RNA samples obtained from 1 ml plasma by ultracentrifugation (100.000 g for 30 min).

### Statistical analysis

The log-rank and Mann-Whitney tests were used for the comparisons of impaired data between different groups of animals and Kaplan-Meier survival analysis of animals with different viral challenges was performed. A commercial Power Analysis and Sample Size (PASS) software (version 2008) (National Computer Systems & Services [NCSS], Kaysville, Utah) was used for statistical power calculation and sample size estimation.

## References

[1] Fauci AS, Johnston MI, Dieffenbach CW, Burton DR, Hammer SM (2008). HIV vaccine research: the way forward.. Science.

[2] Morgan C, Marthas M, Miller C, Duerr A, Cheng-Mayer C (2008). The use of nonhuman primate models in HIV vaccine development.. PLoS Med.

[3] Li J, Lord CI, Haseltine W, Letvin NL, Sodroski J (1992). Infection of cynomolgus monkeys with a chimeric HIV-1/SIVmac virus that expresses the HIV-1 envelope glycoproteins.. J Acquir Immune Defic Syndr.

[4] Shiver JW, Fu TM, Chen L, Casimiro DR, Davies ME (2002). Replication-incompetent adenoviral vaccine vector elicits effective anti-immunodeficiency-virus immunity.. Nature.

[5] Robb ML (2008). Failure of the Merck HIV vaccine: an uncertain step forward.. Lancet.

[6] Watkins DI, Burton DR, Kallas EG, Moore JP, Koff WC (2008). Nonhuman primate models and the failure of the Merck HIV-1 vaccine in humans.. Nat Med.

[7] Wilson NA, Reed J, Napoe GS, Piaskowski S, Szymanski A (2006). Vaccine-induced cellular immune responses reduce plasma viral concentrations after repeated low-dose challenge with pathogenic simian immunodeficiency virus SIVmac239.. J Virol.

[8] Loffredo JT, Maxwell J, Qi Y, Glidden CE, Borchardt GJ (2007). Mamu-B*08-positive macaques control simian immunodeficiency virus replication.. J Virol.

[9] Mothe BR, Weinfurter J, Wang C, Rehrauer W, Wilson N (2003). Expression of the major histocompatibility complex class I molecule Mamu-A*01 is associated with control of simian immunodeficiency virus SIVmac239 replication.. J Virol.

[10] Muhl T, Krawczak M, Ten Haaft P, Hunsmann G, Sauermann U (2002). MHC class I alleles influence set-point viral load and survival time in simian immunodeficiency virus-infected rhesus monkeys.. J Immunol.

[11] Yant LJ, Friedrich TC, Johnson RC, May GE, Maness NJ (2006). The high-frequency major histocompatibility complex class I allele Mamu-B*17 is associated with control of simian immunodeficiency virus SIVmac239 replication.. J Virol.

[12] Joag SV, Stephens EB, Adams RJ, Foresman L, Narayan O (1994). Pathogenesis of SIVmac infection in Chinese and Indian rhesus macaques: effects of splenectomy on virus burden.. Virology.

[13] Ling B, Veazey RS, Luckay A, Penedo C, Xu K (2002). SIV(mac) pathogenesis in rhesus macaques of Chinese and Indian origin compared with primary HIV infections in humans.. Aids.

[14] Marthas ML, Lu D, Penedo MC, Hendrickx AG, Miller CJ (2001). Titration of an SIVmac251 stock by vaginal inoculation of Indian and Chinese origin rhesus macaques: transmission efficiency, viral loads, and antibody responses.. AIDS Res Hum Retroviruses.

[15] Trichel AM, Rajakumar PA, Murphey-Corb M (2002). Species-specific variation in SIV disease progression between Chinese and Indian subspecies of rhesus macaque.. J Med Primatol.

[16] Parker RA, Regan MM, Reimann KA (2001). Variability of viral load in plasma of rhesus monkeys inoculated with simian immunodeficiency virus or simian-human immunodeficiency virus: implications for using nonhuman primate AIDS models to test vaccines and therapeutics.. J Virol.

[17] Champoux M, Higley JD, Suomi SJ (1997). Behavioral and physiological characteristics of Indian and Chinese-Indian hybrid rhesus macaque infants.. Dev Psychobiol.

[18] Clarke MR, O'Neil JA (1999). Morphometric comparison of Chinese-origin and Indian-derived rhesus monkeys (Macaca mulatta).. Am J Primatol.

[19] Doxiadis GG, Otting N, de Groot NG, de Groot N, Rouweler AJ (2003). Evolutionary stability of MHC class II haplotypes in diverse rhesus macaque populations.. Immunogenetics.

[20] Otting N, de Vos-Rouweler AJ, Heijmans CM, de Groot NG, Doxiadis GG (2007). MHC class I A region diversity and polymorphism in macaque species.. Immunogenetics.

[21] Penedo MC, Bontrop RE, Heijmans CM, Otting N, Noort R (2005). Microsatellite typing of the rhesus macaque MHC region.. Immunogenetics.

[22] Viray J, Rolfs B, Smith DG (2001). Comparison of the frequencies of major histocompatibility (MHC) class-II DQA1 and DQB1 alleles in Indian and Chinese rhesus macaques (Macaca mulatta).. Comp Med.

[23] Westmoreland SV, Halpern E, Lackner AA (1998). Simian immunodeficiency virus encephalitis in rhesus macaques is associated with rapid disease progression.. J Neurovirol.

[24] Kestler H, Kodama T, Ringler D, Marthas M, Pedersen N (1990). Induction of AIDS in rhesus monkeys by molecularly cloned simian immunodeficiency virus.. Science.

[25] Pehrson P, Lindback S, Lidman C, Gaines H, Giesecke J (1997). Longer survival after HIV infection for injecting drug users than for homosexual men: implications for immunology.. Aids.

[26] Wolfs TF, de Wolf F, Breederveld C, Sjamsjoedin-Visser LJ, Roos M (1989). Low AIDS attack rate among Dutch haemophiliacs compared to homosexual men: a correlate of HIV antigenaemia frequencies.. Vox Sang.

[27] Ling B, Veazey RS, Hart M, Lackner AA, Kuroda M (2007). Early restoration of mucosal CD4 memory CCR5 T cells in the gut of SIV-infected rhesus predicts long term non-progression.. Aids.

[28] Grabar S, Selinger-Leneman H, Abgrall S, Pialoux G, Weiss L (2009). Prevalence and comparative characteristics of long-term nonprogressors and HIV controller patients in the French Hospital Database on HIV.. Aids.

[29] Jiang X, Wang Y, Ma S (1991). Taxonomic revision and distribution of subspecies of rhesus monkey (macaca mulatta) in China.. Zoological Research (Chinese).

[30] Zhang R, Quan G, Zhao T, Southwick CH (1991). Distribution of macaques (macaca) in China.. Acta Theriologica Sinica.

[31] Robinson TM, Sidhu MK, Pavlakis GN, Felber BK, Silvera P (2007). Macaques co-immunized with SIVgag/pol-HIVenv and IL-12 plasmid have increased cellular responses.. J Med Primatol.

[32] Rerks-Ngarm S, Pitisuttithum P, Nitayaphan S, Kaewkungwal J, Chiu J (2009). Vaccination with ALVAC and AIDSVAX to Prevent HIV-1 Infection in Thailand.. N Engl J Med.

[33] Sodora DL, Allan JS, Apetrei C, Brenchley JM, Douek DC (2009). Toward an AIDS vaccine: lessons from natural simian immunodeficiency virus infections of African nonhuman primate hosts.. Nat Med.

[34] Academies NRCoN (2011). Guide for the care and use of laboratory animals Eighth edition.

[35] Tsai CC, Follis KE, Grant RF, Nolte RE, Wu H (1993). Infectivity and pathogenesis of titered dosages of simian immunodeficiency virus experimentally inoculated into longtailed macaques (Macaca fascicularis).. Lab Anim Sci.

